# Novel Long‐Read Sequencing Method for Characterisation of Hepatitis B Transcripts Show High Expression of Chimeric HBV/Human RNA

**DOI:** 10.1111/liv.70521

**Published:** 2026-02-13

**Authors:** Joakim Bedner Stenbäck, Johan Ringlander, Maria Andersson, Jakob Holm Dalsgaard Thomsen, Sanna Abrahamsson, Gustaf E. Rydell, Magnus Lindh

**Affiliations:** ^1^ Department of Infectious Diseases Institute of Biomedicine, Sahlgrenska Academy, University of Gothenburg Gothenburg Sweden; ^2^ Department of Clinical Microbiology Sahlgrenska University Hospital Gothenburg Sweden; ^3^ Department of Molecular Diagnostics Aalborg University, Hospital Aalborg Denmark; ^4^ Department of Clinical Medicine Aalborg University, Hospital Aalborg Denmark; ^5^ Bioinformatics and Data Centre, Sahlgrenska Academy University of Gothenburg Gothenburg Sweden

**Keywords:** carcinoma, deep sequencing, hepatitis, hepatocellular, transcriptomics

## Abstract

**Background:**

Hepatitis B virus (HBV) genomes integrated into human DNA significantly contribute to surface antigen (HBsAg) production and may drive hepatocellular carcinoma (HCC). Long‐read sequencing methods like Nanopore offer advantages over short‐read next‐generation sequencing (NGS) by providing continuous reads of whole transcripts, but their application to HBV integration analysis remains limited.

**Objective:**

To develop and apply a method combining semi‐nested PCR with Nanopore sequencing to analyse HBV transcripts, including canonical RNA, HBV–human fusion transcripts, and spliced forms in patients with HBV‐ or hepatitis D virus (HDV)‐induced liver disease.

**Methods:**

Nine liver‐transplanted patients with HBV‐ or HDV‐related cirrhosis or HCC were studied. Semi‐nested PCR was used to amplify all HBV transcripts, followed by Nanopore sequencing. The approach allowed differentiation between canonical (cccDNA‐derived) and fusion transcripts. Reads containing the 3′ redundancy beyond nucleotide 1826, exclusive to cccDNA‐derived RNA, were quantified to determine the source of HBV RNA.

**Results:**

Unique and total HBV‐human fusion RNA reads correlated with serum levels of HBV DNA and HBsAg. Integration‐derived RNA accounted for a median of 97% (range: 16%–100%) of HBV RNA. PreS1 RNA levels were much lower than preS2 but sufficient for HDV particle production in an HDV patient without cccDNA‐derived transcripts.

**Conclusion:**

This method enables a simplified and comprehensive analysis of HBV transcripts. The results highlight the predominance of integration‐derived RNA and support the presence of cccDNA‐independent hepatitis D virus production. Nanopore sequencing offers valuable insights into HBV and HDV biology, supporting its role in understanding viral pathogenesis and therapeutic targeting.

## Key Points

- Nanopore long‐read sequencing of HBV RNA provides information about the entire transcripts including HBV–human fusion sites and splicing.
- Over 90% of HBV preS2 reads, but no pregenomic or core reads, were found to be integration‐derived.
- Chimeric HBV–human preS1 transcripts were detected, supporting the presence of cccDNA‐independent hepatitis D virus production.

**Abbreviations**
cccDNA: covalently closed circular DNA
CHB: chronic Hepatitis B
HBeAg: hepatitis B e antigen
HBsAg: hepatitis B surface antigen
HBV: hepatitis B virus
HCC: hepatocellular carcinoma
HDV: hepatitis D Virus
NGS: next‐generation sequencing
pgRNA: pregenomic RNA

## 1 Background

Despite an effective vaccine and antiviral therapy, hepatitis B (HBV) continues to be a significant health concern worldwide, and chronic hepatitis B (CHB) is one of the major causes of cirrhosis and hepatocellular carcinoma (HCC) [1]. The increased risk for HCC is believed to be partly due to integration of the HBV genome into chromosomal DNA in hepatocytes, which may destabilise chromosomal DNA, silence tumour suppressors [2, 3, 4] or enhance oncogene expression [5, 6]. Integrated HBV DNA cannot generate virus particles because the promoter that directs transcription of the HBV pregenomic (pg)/core RNA is not in place [7]. PreS1, preS2 and X RNA can be transcribed from both cccDNA and integrated linear HBV DNA, but only transcripts from cccDNA extend to the conventional HBV poly(A)‐site at approximately nt 1930. Thus, the presence of the sequence between nt 1826–1930 can be used as a marker for cccDNA‐derived transcripts, as opposed to integration‐derived transcripts (preS1, preS2 or X RNA), which have a human 3′ end with variable fusion points, usually upstream of nt 1826 [8].

Third‐generation sequencing platforms such as the MinION from Oxford Nanopore Technologies (ONT) allow ultra‐long reads to be sequenced without prior fragmentation, and facilitate detailed characterisation and minor variant analysis of very long nucleic acids [9, 10]. For HBV this means that entire transcripts may be characterised and that important mutations, such as those associated with HBeAg negativity or antiviral resistance, may be linked to other mutations on the same mRNA. This also enables phylogenetic analysis of the integration‐derived HBV RNA and might allow determination of the timing of integrations. The understanding of when integrations occur during chronic infection, their importance for the production of HBsAg [11], and role in liver cancer is vital for future treatment guidelines and in the end for finding a cure for HBV. The importance of integrations occurring in occult and acute hepatitis also needs further study [12, 13, 14].

Targeted long‐read sequencing using PacBio has been used to investigate HBV integrations [15], but there is a lack of studies applying Nanopore. In this article we present a method for Nanopore sequencing of all HBV mRNAs, including HBV–human chimaeric transcripts. We applied the method on liver tissue from patients with CHB (with cirrhosis and/or HCC) or with anti‐HBc antibodies but without HBsAg indicating cleared HBV infection. We found a high expression from HBV integrations and a positive correlation with HBsAg levels in patient serum in patients with CHB and HCC, but no integrations were detected in patients with previous infection.

## 2 Methods and Materials

### 2.1 Patient Characteristics

In total 12 explant tissue samples from nine HBV‐infected patients were analysed, including tissue from five patients co‐infected with hepatitis D virus (HDV). The mean age of all patients was 56 years (range 35–67 years). Six patients had received treatment with nucleos(t)ide analogues for more than 1 year prior to transplantation. Six patients had detectable HBV DNA in serum at the time of transplantation. The HBV genotype in patients with CHB was A in two, C in one, D in five and E in one. All patients had liver transplantation between 2005 and 2021. Three patients had HCC and two of them contributed with both tumour and non‐tumour tissue; the third only with tumour tissue. Further patient details are found in Table 1.

**TABLE 1:** Characteristics for the patients and proportion of HBV reads that were cccDNA‐derived.

ID	Virus	Age	Gt	HDV RNA^a^	HBsAg^b^	HBV DNA^b^	NUC^c^	Tissue	% of reads that reached beyond nt 1826 (cccDNA‐derived)		
									preS1	preS2	X
1	HBV	55	D	NA	3.79	5.55	No	NT	6.98%	12.3%	8.36%
							No	T		22.52%†	9.77%†
2	HBV	55	D	NA	3.54	2.09	TDF 1 month	NT		39.3%†	29.53%†
								T	1.76%	0.27%†	3.63%
3	HBV	46	C	NA	3.35	1.96	ECV 5 years	T	1.76%†	1.4%†	4.18%†
4	HBV	44	A	NA	4.16	6.47	No	NT	13.2%†	2.81%†	48.4%†
5	HDV/HBV	66	D	6.01	3.43	Negative	ECV 8 months	NT	0%‡	0.16%	0.16%
6	HDV/HBV	53	D	4.99	3.07	Negative	TDF 1.5 years	NT			0%
7	HDV/HBV	54	E	Neg	3.09	1.05	ECV > 3 months	NT	4.28%	0.46%†	38.7%
8	HDV/HBV	47	D	3.94	2.70	Negative	ECV 2 months	NT		1.29%	0.77%
								T		0.00%	37.8%
9	HDV/HBV	35	A	6.89	4.01	1	TDF > 5 months	T		3.28%	3.28%

*Note:* Gt, HBV genotype.

Abbreviations: ECV, entecavir; T, tumour; TDF, tenofovir; Dashes (−) represent an insufficient number of reads to assess transcript origin.

^a^ Log copies/mL at transplantation.

^b^ Log IU/mL at transplantation.

^c^ Nucleoside analogue treatment prior to transplantation. NT, non‐tumour.

^†^ Sample read origin calculated using the total mapped reads instead of the 1750 vs. 1900 method as there was an earlier integration breakpoint than 1750.

^‡^ Sample mapped below 100 reads to transcript and calculations were done manually.

### 2.2 Sample Material and Tissue Homogenization

Liver explant tissue was collected at the operating theatre and put in AllProtect medium (Qiagen, Hilden, Germany) or no medium and frozen in −80°C. Pieces approximately 0.5cm^3^ in size and MagNALyser Green Beads (Roche Diagnostics, Indianapolis, IN, US) were put in 500 μL of lysis buffer (Qiagen) and homogenised using a MagNALyser Oscillator (Roche Diagnostics).

### 2.3 Extraction and DNase Treatment

After RNA extraction with the RNeasy Plus Mini kit (Qiagen), the eluate was treated with Turbo DNase (Invitrogen, Carlsbad, CA, US) and stored at −80°C until PCR.

### 2.4 HBsAg, HBeAg, Anti‐HBc and HBV‐DNA Quantification

HBV DNA levels in serum samples were quantified by Cobas 6800 (Roche Diagnostics). HBsAg, anti‐HBc, HBeAg, and anti‐HBe were analysed using Abbot Architect (Abbott Laboratories, Chicago, IL, USA). HDV RNA was analysed by real‐time PCR using previously described primers and probe [16].

### 2.5 Semi‐Nested PCR and Nanopore Sequencing (SCOPE)

**FIGURE 1:**
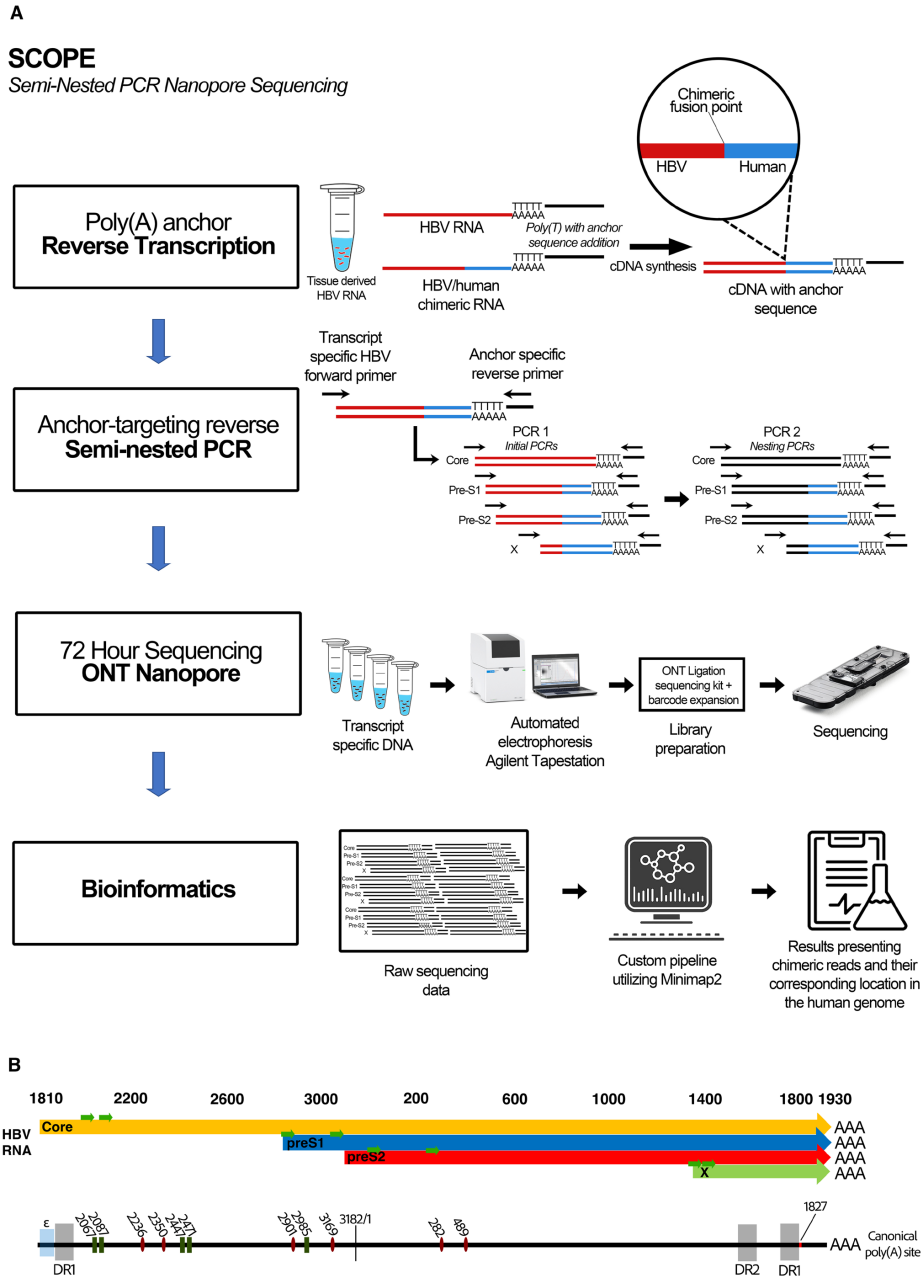
(A) The Nanopore method used for long‐read sequencing of HBV RNA from liver tissue. After reverse transcription with a poly(T) primer with a tag tail, a first PCR was performed with forward primers targeting the HBV transcripts core, pre‐S1, pre‐S2 and X, and a reverse targeting the introduced tag sequence. In a second, semi‐nested PCR step, inner forward HBV primers together with the reverse primer targeting the tag sequence were used. Amplicons were library prepped, barcoded, pooled and sequenced for 72 h on the Nanopore MinIon instrument. A bioinformatic pipeline designed to detect HBV/human reads was applied on the reads. (B) A genomic map of HBV pgRNA showing the location of the most common splicing donors (green rectangles) and splicing acceptors (red ellipses) in relation to viral transcripts. The map also shows the putative integration breakpoint for full‐length double stranded DNA as well as direct repeats, epsilon and the primer location of each transcript (green arrows). *3′ end of dslDNA.

Amplicons for Nanopore sequencing were obtained by semi‐nested PCR, using primers described in Table S1. After a reverse transcription step using a primer with a poly(T) sequence and a tag tail, a first PCR was performed, using forward outer primers with targets in core, preS1, preS2 and X regions of HBV (in separate reactions) in combination with a reverse primer targeting the tag sequence (Table S1) [17]. The second semi‐nested PCR reaction used inner HBV forward primers and a reverse primer targeting the introduced tag. The PCR included an initial denaturation at 95°C for 30 s, and thermocycling with denaturation at 95°C for 15 s, annealing at 55°C for 15 s and an extension for 3 min and 30 s at 72°C for a total of 35 cycles in the first PCR and 20 cycles in the second PCR. A final 6‐min extension step was used in both first and second PCR. The amplicons were subjected to ONT MinION sequencing. Library preparation was performed according to the manufacturer's instructions using the ligation sequencing kit LSK‐109 (Oxford Nanopore Technologies, Oxford, UK). Sequencing was performed on a MK1B MinION platform using R.9.4.1 flow cells in batches of four barcoded and pooled samples and one negative control. An overview of the method as well as a genomic map is presented in Figure 1A,B.

### 2.6 Analysis of PCR Efficiency

The efficiency of the amplification for each target (core, preS1, preS2 and X RNA) was analysed using a full‐length HBV RNA that was constructed in a previous study [18]. A poly(A) tail was attached at the 3′ end of the synthetic RNA using a poly(A)‐tailing kit (Biosearch Technologies, Middlesex, UK) prior to running the same nested RT–PCR as used for the patient samples. The results are presented in the Supporting Information (validation).

### 2.7 Bioinformatics

A custom pipeline utilising Minimap2 read mapping and Python‐based scripts that annotated reads and removed probable spillover reads from other samples produced consensus sequences for each unique integration and compiled integrations found, listing the unique integrations and number of reads covering each HBV–human fusion site. The custom Nanopore pipeline is publicly available at: https://github.com/ClinicalGenomicsGBG/Viral_Integration_Pipeline. An integration was considered unique if > 10 nt distance to a neighbouring HBV/human fusion and if supported by more than five reads. A separate Python script for generating a consensus sequence for each unique HBV transcript was made. Briefly, reads were clustered and one representative HBV sequence was generated for each unique HBV transcript variant with ≥ 10 reads. Unique HBV transcripts were defined as those containing a unique integration site or, if no integration was present, a distinct deletion, splicing event or transcript structure.

The genomic reference used in the analysis was HBV genotype D (AF280817). Additionally, the pipeline output data was used to generate statistical comparisons of chimaeric fusion points, patient HBsAg and HBV DNA levels as well as comparisons between unique integrations and reads among the cohort participants. Splicing and rearrangements were analysed using FLAME [19] after mapping to an HBV pgRNA reference using minimap2. The PCR products of the four different systems were analysed separately. For visualisation of reads, mapping with Minimap2 was performed in CLC Genomics Workbench (v.24, Qiagen).

Coverage graphs were manually examined. Non‐specific amplicons were removed and reads shorter than 150 nt were excluded. Only reads starting at the primer position of every transcript were included to avoid interference from unspecific products.

An additional custom script was made that filtered Nanopore reads for minimum length and starting positions to establish a ratio between cccDNA‐derived and integration‐derived reads, utilising that the 1826–1930 region typically is absent in chimeric HBV–human transcripts. Thus, by comparing the coverage at nt 1750 (which should be included in all transcripts) and nt 1900 (which is present only in cccDNA‐derived transcripts) the proportions of transcripts derived from cccDNA and (by subtraction) from integrations were calculated. For eight samples, the total mapped reads were used instead of the number of reads at nt 1750 to establish transcript origin, since these samples contained earlier integration breakpoints than nt 1750 or premature polyadenylation causing early termination of the transcript. In one sample the number of preS1 reads was insufficient for calculating transcript origin.

### 2.8 Statistical Analysis

Correlation analyses were performed in GraphPad Prism using version 9 or later (GraphPad, San Diego, CA, USA) and JMP (SAS, Cary, NC, USA). A linear mixed model was used in the comparison of the number of unique HBV transcripts in tumour and non‐tumour tissue, as both paired and non‐paired samples were included. Group comparisons of integration reads were made using a linear mixed model. Comparisons of ratios between integration‐derived and non‐integration‐derived HBV RNA based on read coverage were made using Mann–Whitney *U* test.

## 3 Results

### 3.1 Expression of HBV RNA and HBV/Human Chimeric Transcripts

Chimeric HBV–human RNA was detected in all twelve samples from nine patients, as summarised in Table 1. Unique integrations of HBV DNA were defined as integrations identified in the same human gene or non‐coding region in a tissue sample. In all samples, a total of 3758 unique integrations supported by a minimum of 5 reads were identified. On average (median), there were 369 reads (range 5–1768) per integration.

**FIGURE 2:**
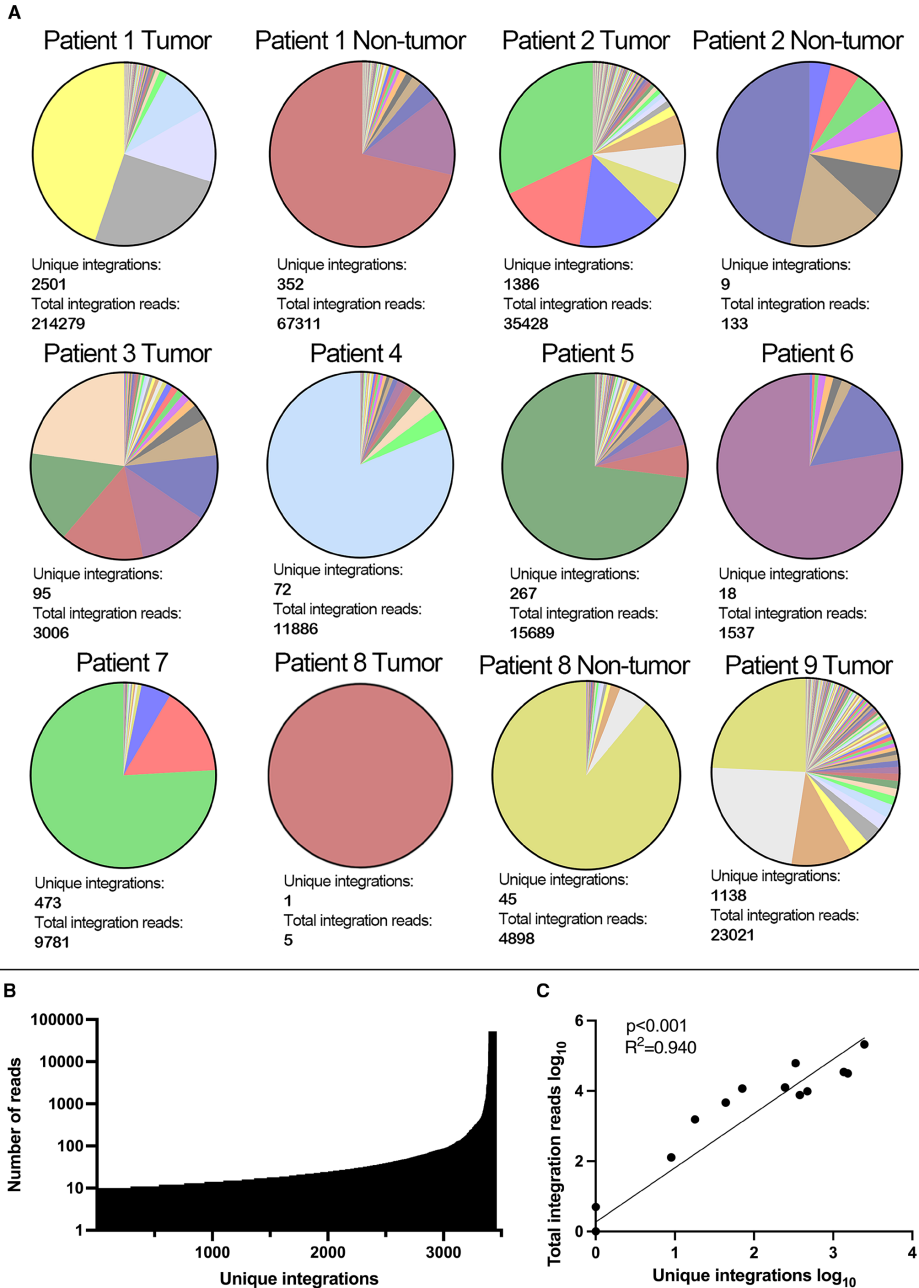
(A) Each unique integration's share of the total number of integration reads, indicating that most of the expression of chimeric HBV/human RNA originates from a few integrations. (B) All unique HBV/Human fusions (x‐axis) sorted lowest to highest number of reads covering the unique integrations, and the number of reads covering each (y‐axis). (C) Correlation analysis between total integration reads and unique integrations (*p* < 0.001).

The proportion of all integration reads that in the individual patients were derived from each unique integration are shown in Figure 2A. The findings indicate that a few transcripts predominated the expression to the extent that in all samples, > 50% of all integration reads were derived from only 1–3 unique transcripts (without any significant difference between tumour and non‐tumour samples) as also illustrated by Figure 2B.

### 3.2 HBV RNA Expression Profiles From cccDNA and Integrations

**FIGURE 3:**
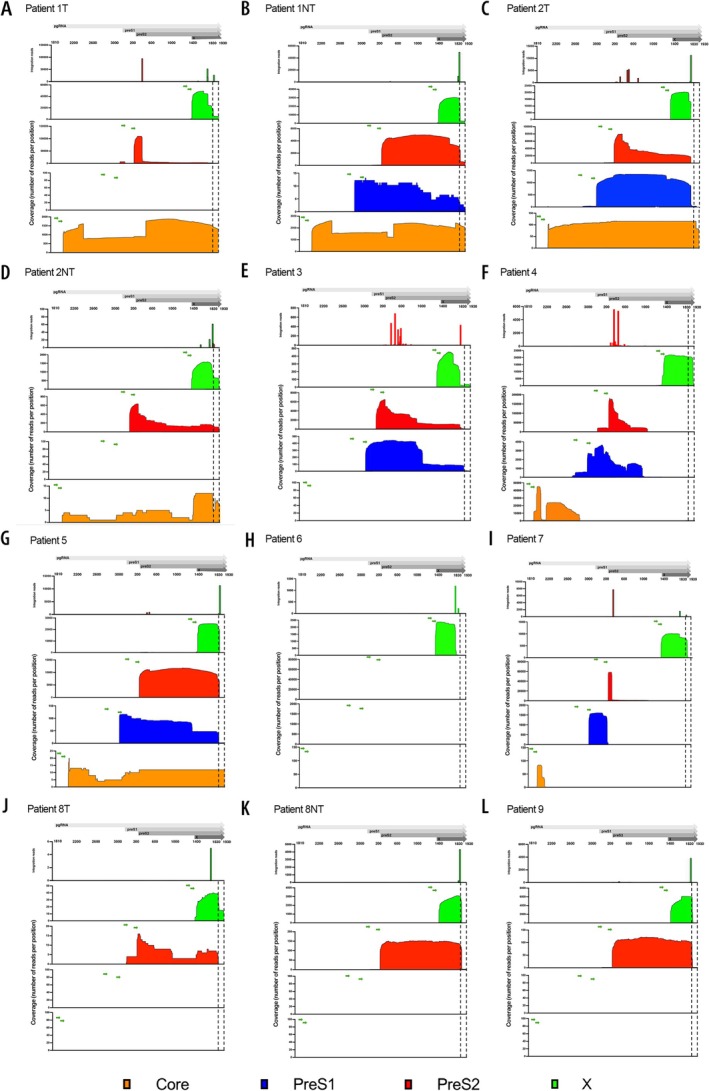
Coverage and integration profiles in explant samples from nine patients. ORFs are shown in the upper panel (grey arrows) with genomic positions listed below it, the short green arrows represent the first and second forward primers of each transcript coded by colour. Presence of reads beyond nt 1826 in the 3′ segment indicates a cccDNA origin of the transcript. The lower coverage in part of core (Patient 1) represents spliced RNA.

Figure 3 shows coverage profiles, i.e., the number of reads from amplicons with forward primers in core, preS1, preS2 or X for all patients. Since the same reverse poly(A) reverse tag was used for all targets, the X PCR amplifies all transcripts, irrespective if the source was cccDNA or integrated DNA. Accordingly, the coverage profile for the X target represents the terminal part of all transcripts, with the exception of transcripts from 5′‐truncated integrations.

**FIGURE 4:**
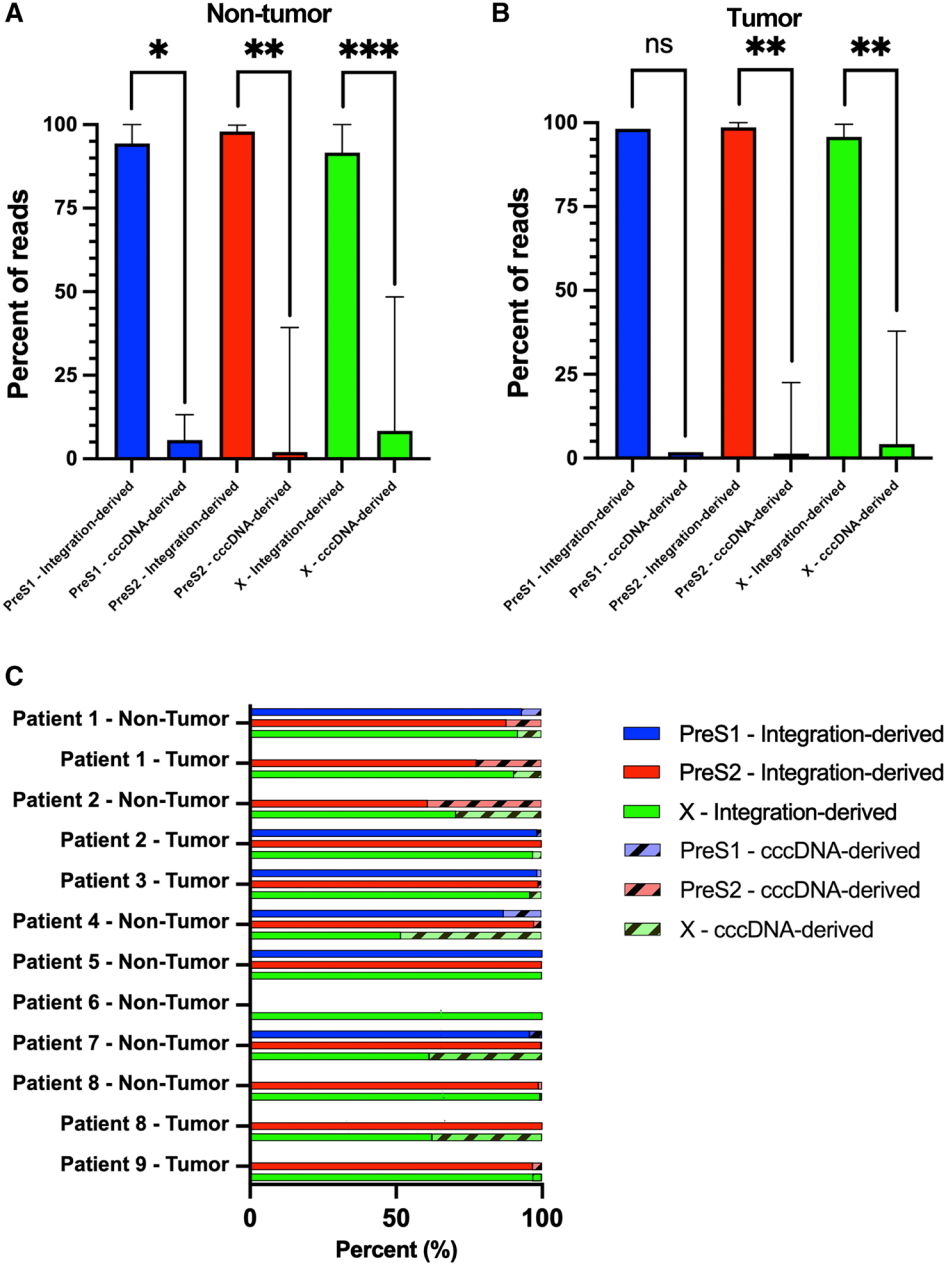
A‐B show the interpreted origin of transcripts and significant difference between median number of cccDNA‐derived and integration‐derived reads for the separate transcripts in (A) non‐tumour and (B) tumour tissue. Comparisons were made using the Mann–Whitney U test. *p* < 0.05 (*), *p* < 0.01(**), *p* < 0.001(***), *p* < 0.0001(****). (C) Percentage of integration‐derived and cccDNA‐derived reads per patient.

As shown in Figure 3 and Figure 4, there was a predominance of expression from integrated DNA for preS1, preS2, and X transcripts, and integration‐derived reads constituted in median 96.7% (range 9.77%–100%) of all reads across all samples and transcripts (Table 1).

To evaluate to what extent amplicon length might influence PCR efficiency we analysed synthetic RNA representing the whole HBV genome (Supporting Information; Validation), and found a 10 times higher efficiency for the X PCR than for the preS1, preS2 and core PCRs. Therefore, comparison of coverages between targets with large difference in amplicon size is not reliable. However, since the difference in amplicon size for the preS1 and preS2 targets is relatively small, the amounts of reads representing these two transcripts were compared, showing a preS2/preS1 ratio of ≈10 in patient 5 and ≈200 in patient 7. Since essentially all HBV RNA from patient 5 originated from integrated DNA, this finding suggests that integrations express preS1 RNA (and accordingly large HBsAg) but with much lower levels than of preS2 RNA.

Most core reads contained the conventional HBV poly(A) site, suggesting that they were expressed from cccDNA as expected. In patient 4, there were a large number of core reads of which the majority terminated between nt 2000 and 2800 without fusion with human DNA or any putative target for the poly(T) primer. A smaller fraction of core reads terminated with poly(A) in the S region where a stretch of A:s (starting at position 920) likely caused by false priming of the reverse primer. Whether this transcript was derived from cccDNA or integrated DNA could not be judged.

The RNA profiles in the tumour samples were highly variable. The samples from patients 3 and 4 showed unusual integration positions in the S region resulting in very short HBV parts of the preS2 chimaeric transcripts (Figure 3E,F). The tumour samples from patients 1 and 2 had significant expression of core RNA (Figure 3A,C), indicating HBV replication in tumour tissue. Tumour tissue from patient 2 showed a premature poly(A) at nt 1808, representing 92% of the reads in preS2 and likewise, non‐tumour tissue showed premature polyadenylation in 27% of the reads in X. Since premature polyadenylated RNA can be derived from either cccDNA or integrated DNA, expression from cccDNA might be underestimated in this sample, although data from one study suggest that premature polyadenylation more often is derived from integrations [15].

### 3.3 Human Poly(A)‐Site in Chimaeric Transcripts

For all samples, the mean length of the human sequence from the fusion point to start of the human poly(A) was as expected highly variable, ranging from 40 to 3623 nt; mean 242 nt for preS1, 170 for preS2 and 211 for X amplicons.

### 3.4 Expression of HBV Transcripts in HDV Infection

Five patients had coinfection with HDV (Figure 3G–L). As shown in Table 1, transcription from integrated DNA was predominant (96.9%–100%) in these cases. A low number of reads in core and spanning the whole genome were detected in patient 5 (Figure 3G). In this patient, also expression of the full preS1 open reading frame was observed and the vast majority of these preS1 transcripts did not extend beyond HBV nt 1826, suggesting expression from integrated HBV DNA.

### 3.5 Splicing and HBV RNA Variants

Splicing was rare in integration‐derived RNA, which was expected since most splicing involves the core region which is not expressed from integrations. Thus, less than 1% of all HBV–human transcripts contained known HBV splicing sites; these sites can be observed in Figure 1B. Splicing was however frequent in samples with pg/core RNA expression (Figure 3), and a median of 11.5% of core RNA amplicon reads showed splicing at conventional sites (ranging 1.76%–18.5%) [20]. The most common splicing variants were SP1, SP9 and SP10, with an expected predominance of SP1. As previously mentioned, there were no significant differences in splicing between tumour and non‐tumour.

**FIGURE 5:**
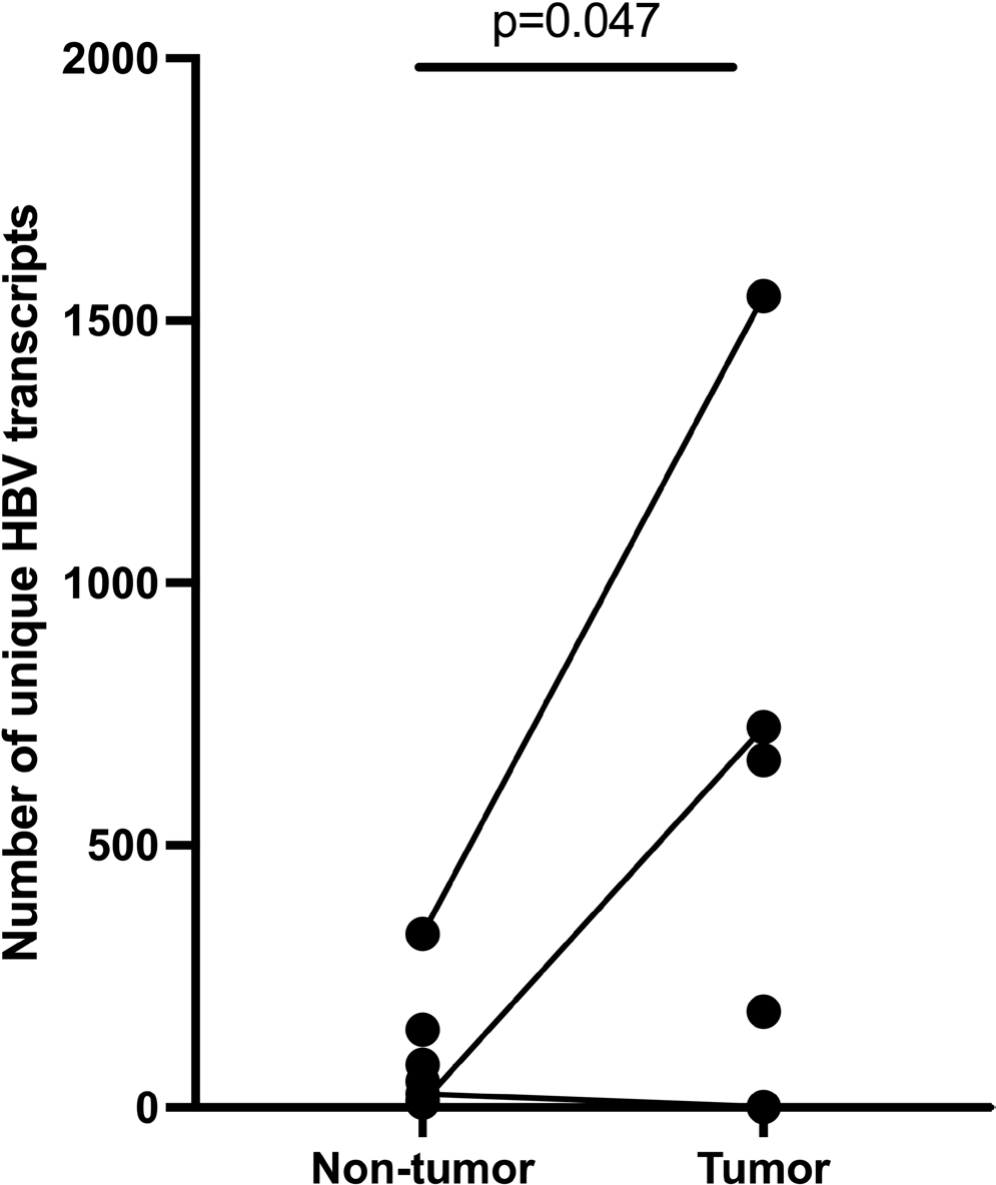
Number of unique transcripts (including chimeric transcripts, deletions and structural variations) in tumour and non‐tumour samples.

The number of unique HBV transcripts, including unique integration‐derived transcripts or different non‐integration‐derived transcripts, was higher in tumour samples compared to non‐tumour samples (mean 625 vs. 94; *t*‐test, *p* < 0.048 linear mixed model), shown in Figure 5. This difference was primarily caused by integration‐derived variants in tumours. The most common breakpoints in the HBV genome in tumour and non‐tumour tissue were nt 414 and nt 1819, respectively. The nt 414 breakpoint stemmed from one tumour sample, harboured 1088 different human chromosomal integration sites with this HBV breakpoint. However, even if this site was excluded, the difference between tumour and non‐tumour remained significant. The most common non‐integration‐derived variant was a deletion of nts 1781–1820, found in three different patients, both in tumour and non‐tumour samples, representing < 1% and 11% of the total unique transcripts, respectively.

### 3.6 Correlation Between Integrations and Serum Markers

**FIGURE 6:**
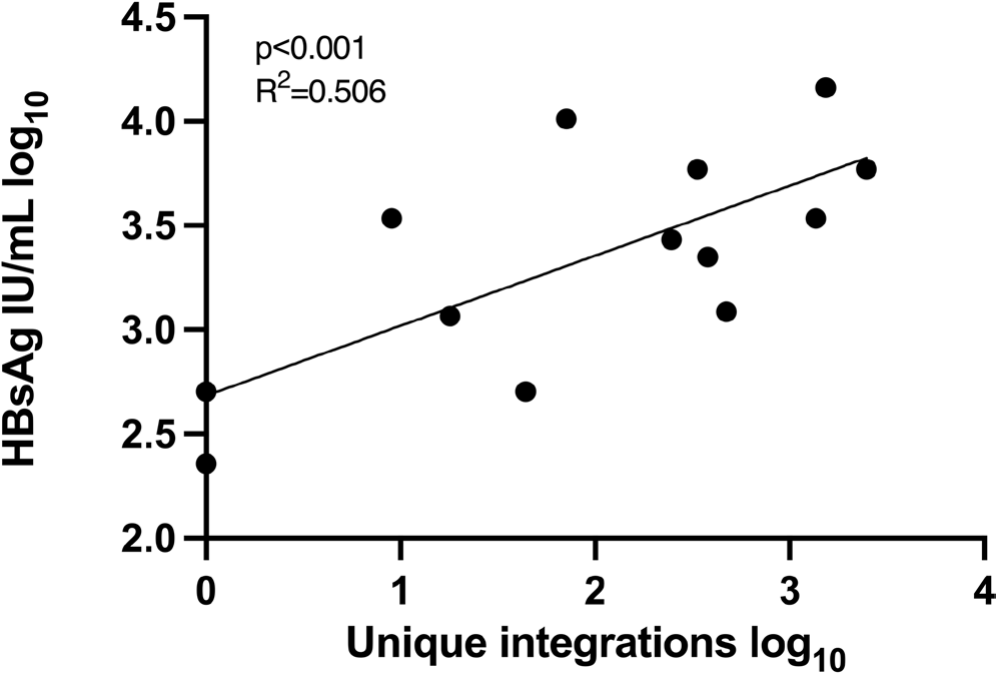
Correlation between number of unique integrations and HBsAg levels in serum (*p* < 0.001).

As shown in Figure 2C, there was a correlation between the number of unique and total integration reads in liver tissue. There was also, as shown in Figure 6, a correlation between unique integrations and serum levels of HBsAg.

## 4 Discussion

This report describes a strategy—SCOPE—using selective PCR, Nanopore long‐read sequencing and automated bioinformatic processing for analysis of HBV transcripts in liver tissue. The results show that most HBV transcripts in liver tissue from patients with cirrhosis due to HBV or HDV are integration‐derived, and they highlight the advantages of Nanopore long‐read sequencing of HBV RNA for identification of integration‐derived transcripts and spliced variants.

The results are consistent with previous short reads‐based studies [21, 22, 23], and as in a previous study [24], we found a large number of unique integrations with very variable expression, strikingly predominated by few of the integrations, and a correlation between the number of unique and total HBV–human chimaeric reads. A main advantage of the long‐read sequencing applied in this study was that we by analysing the 3′ terminal part could determine if HBV transcripts of different lengths were derived from cccDNA or from integrated HBV DNA. This distinction utilises that cccDNA‐derived RNA includes an HBV ‘redundancy’ between nt 1826 and 1930 followed by poly(A), whereas HBV–human chimaeric RNA has a human 3′ terminal part after a fusion point located upstream of nt 1826 in the HBV genome. Thus, the pg/core RNA should always have a 3′ extension because in theory it cannot be transcribed from integrated DNA since the core gene is separated from its promotor [25]. This presumption has been supported by RNA reads profiles observed by short‐read sequencing [11, 21, 26], and by digital PCR quantifications of RNA [27]. However, the long‐read approach in the present study provides direct evidence that core reads contain the 3′ extension between nucleotides 1826 and 1930. Overall, there were fewer reads from the core amplicon compared to preS1, preS2 and X, and no core reads showed an HBV/human fusion site but essentially all had a 3′ extension indicating a cccDNA origin. Reads with this 3′ extension and a conventional poly(A)‐tail at nt 1930 were in minority in most of the reads that started in preS2 or X, indicating that these transcripts mainly were derived from integrations, as previously suggested [21, 27].

In two samples, from patient 7 and patient 4, core reads lacking the 3′ extension were found, but rather than being fused with a human sequence they terminated with a premature poly(A). This might be an artefact due to mispriming during reverse transcription, or reflect premature polyadenylation or an unknown RNA truncation mechanism, as previously discussed by Vachon et al. [28]. Truncated HBV RNAs not stemming from integrations might explain the short reads in samples 2NT and 8 T in which the reads coverage profiles give a (false) impression of integration‐derived RNA.

When applying the SCOPE method on a synthetic HBV RNA (mimicking pgRNA) with poly(A), approximately 4.5% of the amplicons terminated with premature poly(A). In that experiment, mispriming during reverse transcription could not be excluded but was present in less than 4.5%, most likely due to poly(A)‐tails attached by the E. coli PAP to non‐full length synthetic RNA. In HBV RNA from patients, the presence of non‐canonical poly(A) signals in the HBV genome might also provide an explanation [28] rather than mispriming of the poly(T) primer since A‐rich regions were typically not found close to prematurely terminated reads. Further investigation about premature poly(A)‐tails is required, but anyway we conclude that artefacts from reverse transcription should not affect our results more than 4.5%.

Since HBV transcripts have a shared poly(A), reads in the X region represent all these transcripts. By comparing the number of reads present upstream or downstream (nt 1850) of the typical integration sites, we calculated the proportion that was cccDNA derived (reads with nt 1850/reads with nt 1750). We thus found that approximately 90% of all HBV transcripts were integration‐derived. Moreover, both the number of unique integrations and the total number of integration‐derived reads correlated significantly with serum levels of HBsAg, in agreement with previous studies and supporting that integrated HBV DNA is a main source of HBsAg [11, 21, 29].

The long‐read sequencing allowed accurate analysis of spliced HBV RNA. This is of interest because it has been suggested that the presence of spliced variants in serum might be associated with liver cancer or cirrhosis and could potentially be used as a biomarker [30, 31]. We found that spliced HBV RNA in liver tissue was rare in chimeric reads and in reads from preS1, preS2 and X amplicons compared with core RNA, in which spliced RNA constituted approximately 10%. The lack of spliced chimaeric RNA is probably mainly explained by the lack of two of the most common splice donor sites, 2067 and 2087 in integration‐derived transcripts (starting in preS1, preS2 or X) but might also be caused by a different post‐transcriptional RNA processing compared to pg/core RNA [20].

Like preS2 transcripts, most preS1 transcripts lacked an HBV 3′ extension, indicating that they originated mainly from integrations. The number of preS1 reads was much lower than the preS2 reads in most samples (from patients 2, 3, 4 and 5), but such a low level of preS1 RNA might be sufficient to support the formation of HDV particles since their envelope reportedly contains only 1% L‐HBsAg (which is transcribed from preS1 RNA) [32]. The small sample size limits the generalisability of the findings, but we believe that SCOPE is suitable for larger studies to further explore the role of integration‐derived transcripts in both HBV and HDV.

The high number of different integrations in tumour tissue is similar to findings by Zhang et al. and Giosa et al. but differs from others [33, 34, 35]. If tumours originally are mono‐ or oligoclonal, the presence of hundreds or thousands of unique integration sites suggests that tumour cells are susceptible to infection by HBV and may accumulate integrations over time. Alternatively, additional integrations in tumour cells might be generated by chromosomal translocation [15]. Also, infiltration of non‐tumour cells in tumour tissue could be a confounding factor. Clearly, further study of the putative accumulation of integrations in tumour cells is warranted.

A limitation of the method is that the number of reads obtained by the different PCRs can be affected by differences in amplification efficiency. The longer amplicon size of the core PCR, and to some extent the preS1 and preS2 PCR, compared with the X PCR caused an underestimation of the longer transcripts. However, our finding that pg/core RNA and preS1 RNA are less expressed than preS2 and X RNA agrees with data from previous studies using short‐read sequencing without such PCR bias [11, 21, 22]. The observed difference in the number of preS1 and preS2 reads was probably not explained by a lower PCR efficiency as small differences between the number of preS1 and preS2 reads were observed by amplification and sequencing of synthetic full‐length HBV RNA.

An additional limitation is that integration‐derived reads—even though they were abundant—might be underestimated because fusion transcripts with a poly(A) site far downstream of the fusion site would have a lower PCR efficiency and might not even be detected. This possibility is supported by the short observed length (mean 185 nt) of the human sequence past the HBV–human fusion site to the poly(A) site. Finally, an obvious limitation is the small sample size indicating that the findings might not apply to HBV infection in general; therefore, larger studies are warranted to further explore the role of integration‐derived transcripts in both HBV and HDV.

The role of HBV DNA integration has been elucidated in recent years, and it stands clear that integrations contribute to HBsAg production [11, 24, 36]. It has been suggested that integrated HBV DNA might be used as a marker for HCC recurrence after liver resection [37, 38], and the simplified long‐reads sequencing protocol presented in this report could facilitate such applications. Further, long‐read sequencing is preferable over short‐read methods because it allows investigation of entire transcripts including study of mutation linkage, splicing and whether their source is cccDNA or integrated DNA. The simplicity of the Nanopore method and the linked publicly available bioinformatics pipeline makes our method suitable for further study of HBV integration and splicing; similar strategies should be useful also for study of HDV and other viruses.

## Author Contributions

**Magnus Lindh, Johan Ringlander** and **Joakim Bedner Stenbäck** planned and designed the study. **Johan Ringlander, Joakim Bedner Stenbäck** and **Maria Andersson** designed the method. **Johan Ringlander, Joakim Bedner Stenbäck, Jakob Holm Dalsgaard Thomsen** and **Gustaf E. Rydell** performed laboratory work. **Sanna Abrahamsson** designed and performed bioinformatic analyses. **Joakim Bedner Stenbäck** and **Johan Ringlander** drafted the manuscript. All authors contributed to the final version of the manuscript.

## Funding

This work was supported by Cancerfonden, CAN 20 1280 PjF, ALF‐LUA, ALF‐LUA 275108 and the Gothenburg Society for Medicine and Sahlgrenska University Hospital funds.

## Ethics Statement

This study has been granted ethical approval by the regional ethical board in Gothenburg number 853–17.

## Conflicts of Interest

The authors declare no conflicts of interest.

## Supporting information


**Data S1:** Supplementary Information.

## Data Availability

The data that support the findings of this study are openly available in Long‐ read sequencing of HBV/Human chimeric transcripts at https://www.ncbi.nlm.nih.gov/bioproject/?term = PRJNA1085039, reference number PRJNA1085039.
