# Supplementary material for: Novel Long‐Read Sequencing Method for Characterisation of Hepatitis B Transcripts Show High Expression of Chimeric HBV/Human RNA
Source: Liver Int. 2026 Feb 13;46(3):e70521. doi: 10.1111/liv.70521 (PMC12902805; doi:10.1111/liv.70521)
Supplement: Supplementary file 1 — Data S1: Supplementary Information. [file LIV-46-0-s001.docx]

SCOPE Supplementary Material

**Validation of SCOPE**

RNA representing full-lenght HBV was generated as previously described^18^ and ligated with a poly(A) tail. This RNA was used to evaluate PCR efficiency and assess potential mispriming of the poly-T primer. The PCR conditions were identical to those used for patient-derived samples, that is, the same reverse primer was combined with each of four forward primers (core, preS1, preS2 and X) in separate reactions. The amplicons were pooled and subjected to Nanopore sequencing.

PCR with the X forward primer exhibited approximately tenfold greater PCR efficiency than PCR with the other forward primers, as determined by the number of sequencing reads covering each amplicon.

Totally 283 reads terminated with a poly(A) sequence (defined as soft-clipped regions containing at least five consecutive adenosines), likely caused by mispriming of the poly(T) primer and not premature poly(A) sites. These reads represented 1.54 of all 18,430 reads across all amplicons, and 4.54% of the 6,228 reads that terminated prematurely.

**
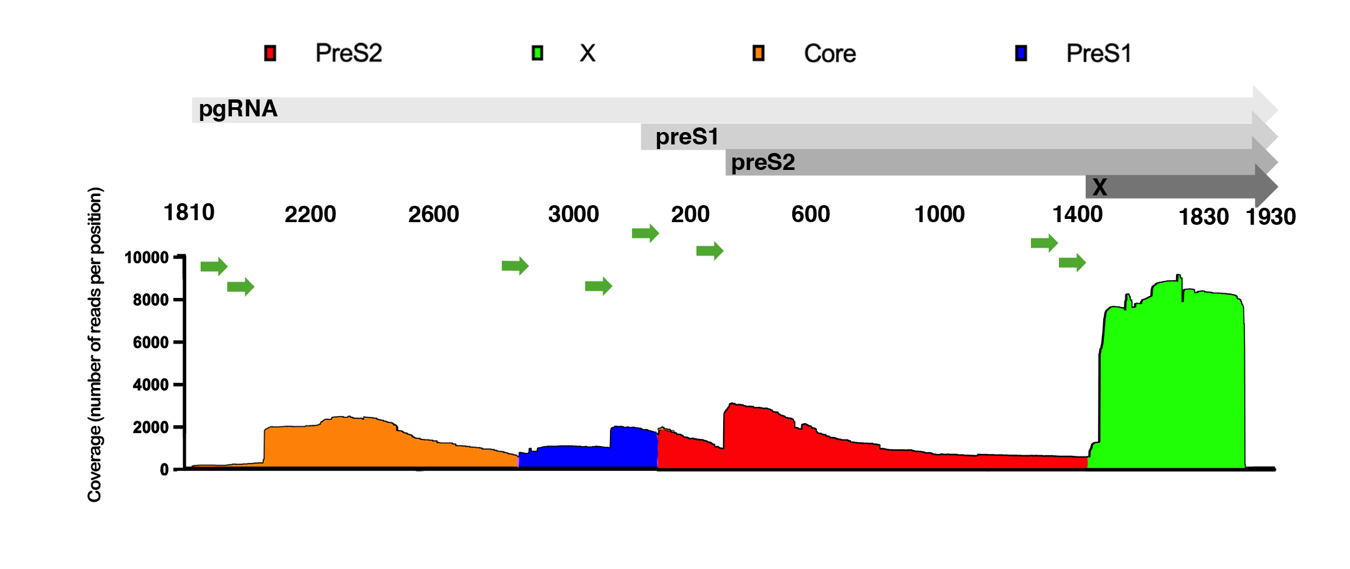
**

**Supplementary Figure 1** A coverage plot showing the results of Nanopore sequencing full-length HBV RNA after PCR amplification using one poly(T) reverse primer and four different forward primers in separate reactions. The long grey arrows show the four transcript start and end, amplicon start and the short green arrows show forward primer positions.

**Supplementary Table 1.** Primers used in the semi-nested PCR

| Primer | Sequence |
| --- | --- |
| Core 1 - 1938 F | GTGGAGTTACTCTCTTTTTTGCC |
| Core 2 - 2049 F | CTCACCATACTGCACTCAAGGCA |
| Pre S1_1 - 2822 F | GTCACCATATTCTTGGGAACAAGA |
| Pre S1_2 - 3131 F | CCTCCTGCCTCCACCAATC |
| Pre S2_1 - 56 F | CCTGCTGGTGGCTCCAGT |
| Pre S2_2 - 251 F | ACTCGTGGTGGACTTCTCTCAA |
| X 1 - 1352 F | TCGCGGAAATATACATCGTTTCC |
| X 2 - 1386 F | TGTACTGCCACCTGGATTCT |
| Anchored Poly(T) A | ACTATTAGAGGGCCGCTTTTTTTTTTTTTTTTA C |
| Anchored Poly(T) G | ACTATCTAGAGCGGCCGCTTTTTTTTTTTTTTTTTG |
| Anchored Poly(T) CA | ACTATCTAGAGCGGCCGCTTTTTTTTTTTTTTTTTCA |
| Anchored Poly(T) CG | ACTATCTAGAGCGGCCGCTTTTTTTTTTTTTTTTTCG |
| Anchored Poly(T) CC | ACTATCTAGAGCGGCCGCTTTTTTTTTTTTTTTTTCC |
